# The role of necroptosis in cancer biology and therapy

**DOI:** 10.1186/s12943-019-1029-8

**Published:** 2019-05-23

**Authors:** Yitao Gong, Zhiyao Fan, Guopei Luo, Chao Yang, Qiuyi Huang, Kun Fan, He Cheng, Kaizhou Jin, Quanxing Ni, Xianjun Yu, Chen Liu

**Affiliations:** 1 Department of Pancreatic Surgery, Fudan University Shanghai Cancer Center, Shanghai, 200032 China; 2 Department of Oncology, Shanghai Medical College, Fudan University, Shanghai, 200032 China; 3 Shanghai Pancreatic Cancer Institute, Shanghai, 200032 China; 4 Pancreatic Cancer Institute, Fudan University, Shanghai, 200032 China

**Keywords:** Necroptosis, Autophagy, Apoptosis, Receptor-interacting protein kinase (RIPK), Mixed lineage kinase domain-like pseudokinase (MLKL), Metastasis, Immunosuppression, Therapeutics

## Abstract

Apoptosis resistance is to a large extent a major obstacle leading to chemotherapy failure during cancer treatment. Bypassing the apoptotic pathway to induce cancer cell death is considered to be a promising approach to overcoming this problem. Necroptosis is a regulated necrotic cell death modality in a caspase-independent fashion and is mainly mediated by ﻿Receptor-Interacting Protein 1 (RIP1), RIP3, and Mixed Lineage Kinase Domain-Like (MLKL). Necroptosis serves as an alternative mode of programmed cell death overcoming apoptosis resistance and may trigger and amplify antitumor immunity in cancer therapy.

The role of necroptosis in cancer is complicated. The expression of key regulators of the necroptotic pathway is generally downregulated in cancer cells, suggesting that cancer cells may also evade necroptosis to survive; however, in certain types of cancer, the expression level of key mediators is elevated. Necroptosis can elicit strong adaptive immune responses that may defend against tumor progression; however, the recruited inflammatory response may also promote tumorigenesis and cancer metastasis, and necroptosis may generate an immunosuppressive tumor microenvironment. Necroptosis also reportedly promotes oncogenesis and cancer metastasis despite evidence demonstrating its antimetastatic role in cancer. In addition, necroptotic microenvironments can direct lineage commitment to determine cancer subtype development in liver cancer. A plethora of compounds and drugs targeting necroptosis exhibit potential antitumor efficacy, but their clinical feasibility must be validated.

Better knowledge of the necroptotic pathway mechanism and its physiological and pathological functions is urgently required to solve the remaining mysteries surrounding the role of necroptosis in cancer. In this review, we briefly introduce the molecular mechanism and characteristics of necroptosis, the interplay between necroptosis and other cell death mechanisms, crosstalk of necroptosis and metabolic signaling and detection methods. We also summarize the intricate role of necroptosis in tumor progression, cancer metastasis, prognosis of cancer patients, cancer immunity regulation, cancer subtype determination and cancer therapeutics.

## Background

It is well-established that apoptosis, which is a programmed cell death mechanism, functions as a natural barrier that protects against cancer development [1]. However, the evasion of and resistance to apoptosis are also considered indisputable hallmarks of cancer [1], and resistance to apoptosis is often responsible for both tumorigenesis and drug resistance, resulting in chemotherapy failure [2]. In addition to overcoming apoptosis resistance, developing approaches to induce nonapoptotic forms of programmed cell death as alternative therapeutics in cancer is imperative and attractive.

Apoptosis has historically been believed to be the only form of programmed cell death (PCD), and necrosis, which was believed to be an “accidental” type of death not regulated by molecular events [3], was assumed to be the diametrically opposite modality of cell death compared to apoptosis until necroptosis was discovered as a novel programmed form of necrotic cell death that bears a mechanistic resemblance to apoptosis and a morphological resemblance to necrosis [4]. Necroptosis is mainly mediated by RIPK1 (receptor-interacting protein [RIP] kinase 1), RIPK3, and MLKL (mixed lineage kinase domain-like pseudokinase) and characterized to be inhibited by the necrostatin-1 (Nec-1), which is the first well-defined necroptosis inhibitor that exclusively inhibits RIPK1 activity [5].

In addition to its key role in viral infection and development, necroptosis has been suggested to play a pivotal role in the regulation of cancer biology, including oncogenesis, cancer metastasis, cancer immunity, and cancer subtypes [6, 7]. As a coalescence of apoptosis and necrosis, the following dual effects of necroptosis on cancer have been demonstrated: on the one hand, the key mediators of the necroptotic pathway alone or combined have been suggested to promote cancer metastasis and cancer progression [8–10]; however, on the other hand, necroptosis also reportedly serves as a “fail-safe” mechanism that protects against tumor development when apoptosis is compromised [11, 12]. Considering the pivotal role of necroptosis in cancer biology, necroptosis emerged as a novel target for cancer therapy, and a growing arsenal of compounds and multiple therapeutic agents reportedly defend against cancer by inducing or manipulating necroptosis [13].

## Overview of the molecular mechanism of necroptosis

Because necroptosis has increasingly been considered important in cancer, a deeper understanding of the mechanisms of necroptosis is essential for developing a novel approach to regulate necroptosis in cancer Table 1.

**Table 1:** Key mediators in necroptosis and their key function

Key Mediators	Function in necroptosis	Inhibitors	Reference
RIP1	determining the survival or death of cell; recruiting and activating RIPK3 to form necrosome	nec-1	[5, 14, 15]
RIPK3	interacting with RIPK1 to form necrosome; phosphorylating MLKL	GSK843 and GSK872	[15, 16]
MLKL	phosphorylated by RIPK3; oligomerized and translocated to plasma membrane to execute necroptosis	NSA	[16]
cIAP1/2	polyubiquitinating RIPK1 to induce NF-κB signaling	smac mimetics	[14, 19]
CYLD	deubiquitinating RIPK1; promoting “Ripoptosome” formation; promoting necrosome formation	–	[20]
caspase-8	cleaving RIPK1 and RIPK3 and activating apoptosis; inhibiting necrosome formation; cleaving CYLD to promote cell survival	zVAD-fmk	[21, 23, 24]

Theoretically, a plethora of different stimuli, including members of the tumor necrosis factor receptor (TNFR) superfamily, pattern recognition receptors (PRRs), T cell receptors (TCRs) and multiple chemotherapeutic drugs, can activate the necroptotic cell death pathway [25]. Environmental stresses such as hypoxia can also elicit necroptosis [26], which reportedly may be abolished by glucose uptake and enhanced anaerobic glycolysis in cancer cells [26]. Among the various stimuli, the TNFα/TNFR signaling pathway is considered a prototype and has been the most intensively investigated [14]. Thus, the initiation of necroptosis can be epitomized by the events occurring in the TNF signaling pathway. The binding of TNF to TNFR1 induces a conformational change in TNFR1 trimers, leading to the recruitment of multiple proteins, including RIPK1, TRADD (TNFR-associated death domain), cIAP1 (cellular inhibitor of apoptosis protein 1), cIAP2, TRAF2 (TNFR-associated factor 2) and TRAF5, by TNFR1. This membrane-bound multimeric protein complex is named complex I [27], and within this complex, RIPK1, which is a crucial regulator of cell fate [15], is polyubiquitinated by cIAP1/2, which, in turn, induces the canonical NF-κB (nuclear factor kappa B) pathway [14], which transactivates cytoprotective genes and facilitates cell survival [27].

Furthermore, due to the rapid internalization of ligand- bound TNFR, the proteins in complex I and their posttranslational modification are consequently altered [14]. For instance, RIPK1 is deubiquitinated by the deubiquitinase cylindromatosis (CYLD), which subsequently limits the sustained activation of NF-κB signaling [20] and leads to a tendency towards the activation of cell death pathways. Consequently, a cytoplasmic death-inducing signaling complex comprising RIPK1, TRADD, caspase-8 and FADD (FAS-associated death domain protein), which is known as complex II and is also referred to as “Ripoptosome” [28], is formed, inducing caspase-8 activation [29]. Complex II is involved in the activation of both apoptotic and necroptotic pathways. In complex II, active caspase-8 cleaves both RIPK1 and RIPK3, resulting in their inactivation, and the proapoptotic caspase activation cascade is initiated, ultimately leading to apoptosis execution [23]. Caspase-8 is also reported to promote cell survival by cleaving CYLD [21]. However, following the inhibition of caspase-8 due to pharmaceutical or genetic intervention [27], RIP kinases cleavage stops, and the cell death pathway is directed to necroptosis.

After the cell death mode is switched, RIPK1 is phosphorylated through the autophosphorylation of the serine residue 161(S161) at its N-termini and is, thus, activated [5]. Activated RIP1 interacts with RIPK3 through their ﻿RIP homotypic interaction motif (RHIMs) [15], leading to the formation of a heterodimeric amyloid structure named the necrosome complex, which is a key molecular signaling platform in necroptosis primarily comprising RIPK1 and RIPK3 [15]. Mitochondrial reactive oxygen species (ROS) was found to activate RIPK1 autophosphorylation, leading to RIPK3 recruitment, and ROS induction also requires RIPK3 in necrosome, thus forming a positive feedback circuit in which necroptosis is induced effectively [30]. CYLD was reported to promote necrosome formation and activation by deubiquitinating RIPK1 after necrosome assembly [22]. Necrosome formation and/or activation can be blocked by RIPK1 inhibitor nec-1, MLKL inhibitor necrosulfonamide (NSA) and multiple RIPK3 inhibitors [31].

**Fig. 1:**
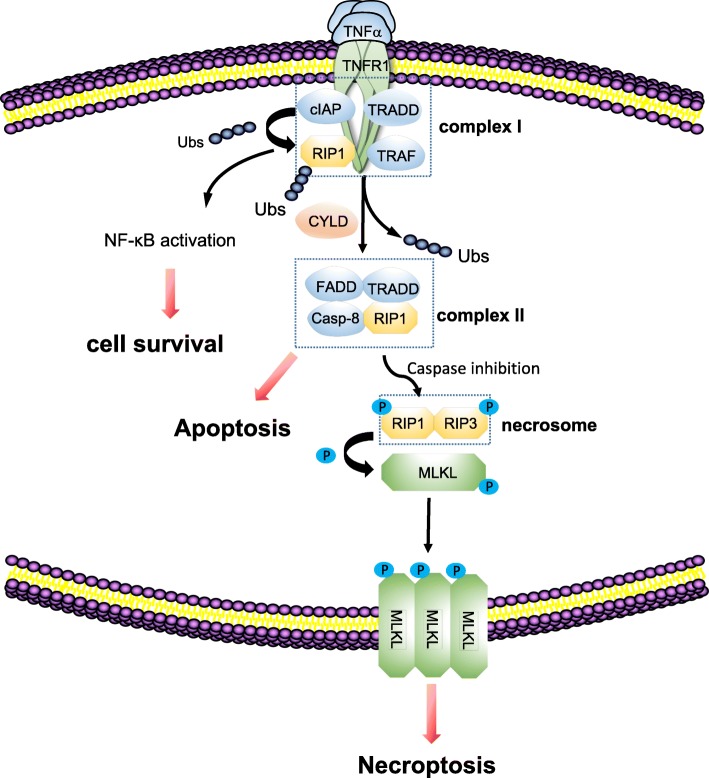
TNF necroptosis signaling mechanism

In necrosomes, RIPK3 phosphorylates its well- characterized functional substrate MLKL. MLKL is then oligomerized and translocated to the plasma membrane, thus leading to the execution of necroptosis, causing necrotic plasma membrane permeabilization and ultimately cell demise characterized by the swelling of the cell and loss of the cell and organelle integrity [16, 32, 33] (Fig. 1)*.*

The binding of TNF to TNFR1 leading to the recruitment RIPK1, TRADD, cIAP1 and cIAP2, TRAF2 and TRAF5 and thus the formation of complex I. When RIPK1 is polyubiquitinated by cIAP1/2, NF-κB pathway is activated and cell survival prevails. When RIPK1 is deubiquitinated by CYLD, NF-κB pathway was limited and complex II composed of RIPK1, TRADD, caspase 8 and FADD was formed. Caspase 8 inactivates RIPK1 and RIPK3 by proteolytic cleavage, which leads to apoptosis pathway. When caspase 8 is inhibited, a crucial complex, necrosome, is formed, in which RIPK3 phosphorylates its substrate MLKL, leading to the its oligomerization and its translocation to plasma membrane to execute necroptosis, causing necrotic plasma membrane permeabilization and ultimately cell death associated with loss of cell and organelle integrity.

## Comparison of the key features of necroptosis and apoptosis

While necroptosis is characterized by caspase- independence, it bears similarity to and shares a part of the molecular pathway of apoptosis, particularly the extrinsic apoptotic pathway; however, necroptosis remarkably varies in the morphological and immunological outcomes. Necroptosis exhibits morphological features similar to necrosis, including the rupture of the cellular membrane, ﻿progressively translucent cytoplasm and swelling of organelles [27, 34]. In contrast, the morphological features of apoptosis are typified by membrane blebbing, cellular shrinkage, nucleus fragmentation and chromatin condensation [35]. The rupture of the plasma membrane in necroptotic cells leads to the release of cell contents, which can cause the exposure of damage-associated molecular patterns (DAMPs) and trigger robust inflammatory responses [36], indicating that necroptotic cells are markedly more immunogenic than apoptotic cells where latent DAMPs are confined to the relatively intact plasma membrane or are encased in apoptotic bodies [36], and their corpses are engulfed and dissolved by phagocytes, suggesting that apoptosis typically does not induce an immune response as robust as that induced by necroptosis [37]. Necroptosis was also found to be involved in the maintenance of T cell homeostasis [36] because ﻿necroptosis has been indicated to clear up excessive and abnormal T cells in the absence of caspase-8 [38], which is considered to aid apoptosis by acting as the major machinery countering the abnormal proliferation of lymphocytes [39].

## Interplay among necroptosis, apoptosis and autophagy

Although the signaling pathways of each of the three cell death modes have been well elucidated, cell death in vivo is often characterized by their intricate interplay. Understanding the interrelationship among the three forms of PCD is pivotal for manipulating their synergistic and antagonistic effects in anti-cancer therapies and developing novel approaches to target the converging point of the cell death pathways.

In most settings, apoptosis is the default cell death modality, whereas necroptotic pathway is generally viewed as a “fail-safe” cell death machinery occurring when key apoptotic mediators are blocked by pharmacological inhibition or genetic ablation or in cases in which stressed cells are unable to undergo apoptosis. However, in the context of certain viral infections, such as ﻿vaccinia virus [40], or when induced by certain compounds, such as shikonin [41], necroptosis may predominate as the cell demise mode. Furthermore, studies have indicated that the intracellular adenosine triphosphate (ATP) level may switch the cell death decision as follows: ATP depletion prevents apoptosis and induces necrosis, and glucose ﻿replenishment in ATP- depleted T cells converts the cell death pathway to apoptosis because ﻿apoptosis is a highly energy-consuming process principally involving ATP- requiring steps, such as caspase activation and apoptosome formation [42, 43]. Thus, the energetic state of cells may also influence the cell death modality.

Autophagy is a “self-eating” process that provides nutrients and energy under various stresses, including starvation, cellular and tissue remodeling, and cell death, by degrading cytoplasmic proteins and organelles within lysosomes [44, 45] and is also regulated by cellular energy availability [46]. Autophagy reportedly saves ATP- depleted cells from ﻿necrosis/necroptosis by restoring energy, and the inhibition of autophagy may incur a metabolic crisis and promote necroptosis [46, 47]. Necroptosis has also been reported to promote autophagy. For instance, the high level of reactive oxygen species (ROS) generated during necroptosis may cause the induction of autophagy, which is responsible for degrading damaged organelles and proteins. Moreover, RIPK1 has been suggested to play a significant role in the modulation of autophagic signaling, which is independent of necroptosis [48].

Similarly, in most cases, autophagy inhibits the initiation of apoptosis, and the activation of caspases in the apoptotic pathway leads to the cleavage of key pro- autophagic mediators [49]. In some cases, autophagy may also promote apoptosis by eliminating endogenous inhibitors of apoptosis or creating a platform for capsapse-8 activation via autophagosome formation [49]. ﻿Goodall et al. showed that in the background of Map3k7 deletion, the autophagic pathway may switch the cell death mode to from apoptosis to necroptosis by ﻿acting as a scaffold allowing the necrosome to be more efficiently activated, which is mediated by the p62-dependent recruitment of RIPK1 to the autophagic machinery [50]. When the mechanism is blocked, the cell may die through apoptosis [50].

﻿Evidently, the interplay among apoptosis, necroptosis and autophagy is profoundly intricate and requires further exploration.

## Crosstalk of necroptosis and metabolic signaling

Necroptotic pathway and its key regulators have been implicated in metabolic signaling. RIPK3 has been reported to activate key enzymes in metabolic pathways, including glycogen phosphorylase (PYGL) [51], a vital enzyme in utilizing reserved glycogen as an energy source, and pyruvate dehydrogenase (PDH) [52], the key enzyme that links glycolysis to aerobic respiration, resulting in enhancement of glycolysis and aerobic respiration and eventually leading to increased ROS generation. Furthermore, the increase of aerobic respiration mediated by RIPK3 in necrosome positively feeds back on necrosome formation via ROS [53]. Moreover, RIPK3 can also promote the activity of glutamate-ammonia ligase (GLUL) and glutamate dehydrogenase 1 (GLUD1), two enzymes involved in glutaminolysis, which may as well contribute to elevated ROS generation [51]. RIPK1 was shown to downregulate the activity of adenine-nucleotide translocators (ANT) [54], a mitochondrial enzyme responsible for the trading ADP for ATP [55], suggesting the increased RIPK1 activity during necroptosis may also augment the production of ROS.

Huang et al. have reported that GLUT-dependent glucose uptake and glycolytic metabolism may inhibit resistance to hypoxia-induced RIPK signaling and necrotic features in colorectal carcinoma cells, and that glycolytic pyruvate can revert hypoxia-induced necroptosis probably through ﻿mitochondrial ROS scavenging [26]. The study further highlighted the interrelationship between necroptosis and metabolism, and indicated that potential targeting therapeutics glucose and pyruvate may overcome the barrier caused by enhanced angiogenesis and metastasis driven by hypoxic stress in cancer treatment [26].

McCaig et al. [56] has recently reported that hyperglycemia may cause an shift from extrinsic apoptosis to RIP1-dependent necroptosis in both human primary T cells and monocytes, which is dependent on glycolysis and its production of mitochondrial ROS. This work has demonstrated that hyperglycemia may incline cells to undergo necroptotic pathway in spite of the initial activation of apoptosis, and has further elucidated the role of metabolic condition in regulating cell death mode.

## Identification of necroptosis

Because no specific molecular markers for necroptosis detection are available to date, identification of necroptosis often requires a combination of methods of detection. In cultured cells, transmission electron microscopy (TEM) can be used to identify the necrotic morphology [57]. The detection of necroptosis via biomarkers has been principally focused on the key molecular events involved in necroptosis [58], including the activation of RIPK1, RIPK3 and MLKL, the formation of the necrosome, and MLKL oligomerization and membrane translocation [58].

The biomarkers used to detect the activation of RIPK1, RIPK3, and MLKL in necroptosis include phosphorylated RIPK1/3 and MLKL at their phosphorylation site, which are detected mainly by using their corresponding anti- phospho-ser/thr antibodies in a western blot analysis (WB) [59]. The biomarker used to detect the necrosome formation is the RIP1/RIP3 complex, which is an amyloid- like structure and is detected by immunoprecipitation [51] and electron microscopy image analysis [59]. MLKL oligomerization and membrane translocation are detected by WB and immunostaining analyses, respectively [33, 60].

﻿Several pharmacological inhibitors, such as the RIPK1 inhibitor necrostatin-1 (Nec-1), the MLKL kinase inhibitor necrosulfonamide (NSA) [16] and the RIPK3 inhibitor GSK843 and GSK872 [16–18], antagonize the necroptotic pathway and can recue cells from necroptotic cell death; these inhibitors have also been used to detect necroptosis.

Necroptosis identification in vivo is also problematic. The induction of necroptosis in vivo is indicated by elevated mRNA or protein levels of RIPK1, RIPK3 or MLKL [61]. In transgenic animal models in which RIPK1, RIPK3 or MLKL are genetically knocked out or following blockade using respective chemical inhibitors, ﻿necroptosis can be identified by reduced cell demise or tissue injury [61].

Furthermore, recent evidence suggests that in some cases, RIPK1 is not required for the necroptotic pathway. For instance, Kaiser et al. [62] demonstrated that Toll like receptor 3/4 (TLR-3/4) induced necroptosis in certain cells is independent of RIPK1 but still depends on both RIPK3 and MLKL. These findings suggest that RIPK3 and MLKL are more specific molecule biomarkers for necroptosis identification.

## Relevance of necroptosis in Cancer

Necroptosis has been reported to be both a friend and a foe of cancer; its dual effects of promoting and reducing tumor growth have been found in different types of cancer. As a fail-safe form of cell death occurring in cells in which apoptosis fails to be induced, necroptosis can prevent tumor development. Nevertheless, as a necrotic cell death modality, necroptosis can trigger inflammatory responses and reportedly promotes cancer metastasis and immunosuppression [63, 64].

### Expression of Necroptotic factors and its influence on prognosis in Cancer

The downregulation of the expression of numerous key molecules in necroptotic signaling pathways has been found in different types of cancer cells, suggesting that cancer cells may evade necroptosis to survive (Table 2)*.*

**Table 2:** Expression of necroptotic factors in cancer and its influence on cancer prognosis

Cancer Type	Expression of Necroptotic Factors	The Influence on Prognosis	Reference
Breast Cancer	decreased RIPK3 expression	worse prognosis	[7, 65]
Colorectal Cancer	decreased RIPK3 expression; decreased MLKL expression	reduced DFS and OS; reduced OS	[12, 66, 67]
Acute Myeloid Leukemia	decreased RIPK3 expression	accelerated leukemogenesis and worse survival	[11, 68]
Melanoma	decreased RIPK3 expression; decreased CYLD expression	not mentioned; enhanced tumor progression and metastasis	[69, 70]
Head and Neck Squamous Cell Carcinoma	decreased RIPK1 expression	enhanced tumorigenesis	[9]
Chronic Lymphocytic Leukemia	decreased CYLD expression	reduced OS	[71]
Glioblastoma	increased RIPK1 expression	worse prognosis	[10]
Lung Cancer	increased RIPK1 expression	promoted oncogenesis	[72]
Pancreatic Cancer	increased expression of RIPK1, RIPK3, FADD and MLKL	promoted oncogenesis	[64, 73]
Gastric Cancer	decreased MLKL expression	reduced OS	[74]
Ovarian Cancer	Decreased MLKL expression	reduced OS	[75]
Cervical Squamous Cell Carcinoma	decreased MLKL expression	reduced OS	[76]

RIPK3 expression is absent or decreased in numerous cancer cell lines [65, 69]; specifically, the loss of RIPK3 protein expression was found in two-thirds of the 60+ cancer cell lines tested [65]. A decreased RIPK3 expression has also been reported in samples from human patients with cancer, such as breast cancer [7, 65], colorectal cancer [12, 66], acute myeloid leukemia (AML) [11, 68] and melanoma [69]. Moreover, Hockendorf et al. [11] reported that leukemogenesis was markedly accelerated following the knockout of RIPK3 in mice transplanted with bone marrow cells bearing a mutated AML driver gene and that the survival of the RIPK3 knockout mice was poorer than that of the wild-type mice. In addition, the tumor-suppressing effects of RIPK3 have been documented in colorectal cancer. In a cohort study involving more than one hundred patients, low RIPK3 expression was found to independently prognosticate a reduced DFS (disease-free survival) and OS (overall survival) [12]. Furthermore, RIPK3 knockout mice were reportedly at a higher risk of developing colitis-associated colorectal cancer and producing a higher number of pro-inflammatory or tumor-promoting factors [77]. Similarly, low RIPK3 expression indicates a worse prognosis in patients with breast cancer [65]. These studies suggest that RIPK3 might play an anti-inflammatory and antitumor role in cancer. Furthermore, reports indicate that genomic methylation and/or hypoxia may play a pivotal role in silencing RIPK3 expression in many cancer cell lines [26, 65, 66].

Consistent with these observations, McCormick et al. [9] reported that RIPK1 expression is downregulated in head and neck squamous cell carcinoma as well; this downregulation was proven to be correlated with disease progression. The authors suggested that the downregulation of RIPK1 expression promoted by epigenetic changes during tumor progression enables tumor cells to evade anoikis, which may stimulate tumorigenesis by enhancing the metastatic abilities of the tumor cells [9]. In addition, the expression of CYLD, which is a deubiquitinating enzyme that is a key mediator in the necroptotic pathway, was found to be decreased in chronic lymphocytic leukemia (CLL) [71] and malignant melanoma [70]. In CLL, low CYLD expression identifies a subgroup of patients with worse OS [71]. In melanoma, the repression of CYLD by the transcription factor Snail1 contributes to cell proliferation and cancer invasiveness in vitro and tumor progression and metastasis in vivo [78]. However, because CYLD is also involved in the NF-kB pathway, which mediates inflammation and tumor growth [79], the effects of CYLD downregulation may not only be associated with necroptosis.

Collectively, these studies implicate the antitumor role of the necroptosis pathway in cancer. However, the downregulation of necroptotic factors does not appear to occur in all cancers; the expression of necroptotic factors has been found to be upregulated in some cancers. For example, in glioblastoma, RIPK1 is commonly overexpressed, and the upregulation of RIPK1 expression is correlated with a poorer prognosis [10]. Similarly, RIPK1 expression is markedly elevated in both human lung cancer samples and mouse lung tumor models, and RIPK1 has been suggested to play an oncogenic role [72]. Notably, the expression of RIPK1, RIPK3, FADD and MLKL is elevated in pancreatic ductal adenocarcinoma (PDA) [64], which is accompanied by accelerated oncogenesis.

Interestingly and counterintuitively, according to Colbert et al. [73] the decreased expression of MLKL was correlated with a decreased OS in patients with early- stage resected pancreatic adenocarcinoma. Moreover, the reduced level of MLKL was markedly correlated with the reduced OS in gastric cancer [74], ovarian carcinoma [75], cervical squamous cell carcinoma [76] and colon cancers [67], probably because MLKL can affect the modulation of local tumor microenvironment immunosurveillance. These findings suggest that MLKL is a candidate prognostic biomarker in those cancers.

### Necroptosis and Cancer Immunosurveillance

The immunosurveillance of cancer refers to the process by which the immune system identifies and eliminates cancerous and/or precancerous cells based on tumor-specific antigens (TSAs) or tumor-associated antigens (TAAs) [80] before these cells constitute a threat to our health [81]. This process is mediated by innate and adaptive immune cells and effector molecules, including dendritic cells (DC), cytotoxic T cells, ﻿M1 macrophages, natural killer (NK) cells, natural killer T (NKT) cells and their corresponding cytokines [81, 82]. RIPK3 has been found to be required in the regulation of cytokine expression in DCs, which are crucial sentinels that regulate immune homeostasis by expressing modulatory cytokines and interlinking the innate and adaptive immune systems [83]. Additionally, despite evidence indicating that RIPK3 signaling may not play a role in the regulation of the activation of T lymphocytes, B lymphocytes and macrophages [84], RIPK3 has been suggested to regulate NKT cell function and promote the NKT cell- mediated anti-tumor immune response by activating mitochondrial phosphatase phosphoglycerate mutase 5 (PGAM5) through a process that is independent of the necroptosis pathway [85]. Additionally, while the function of apoptosis in the maintenance of central tolerance has been well defined, several reports have indicated that necroptosis plays a regulatory role in the antigen-induced proliferation of T cells mainly via the elimination of excessive T cells, which is essential for maintaining homeostasis in peripheral T cells and the survival of T cells when they are activated by stimuli, and necroptosis-dependent process is negatively regulated by caspase-8 [86]. Necroptosis reportedly occurs during the late stage of T-cell proliferation and necroptotic signaling is markedly intensified in T cells absent in FADD, suggesting that FADD may negatively regulate necroptosis mediated by T cell receptors [87].

In addition to the direct interactions with immune cells, necroptosis initiates adaptive immune responses by releasing DAMPs into the tissue microenvironment [37], and after necroptotic cells are phagocytosed, phagocytic cells, such as DCs and macrophages, can release pro- inflammatory cytokines that increase stimulating molecules and amplify cross-presentation, incurring strong immune responses [36, 88]. Necroptotic cells can provide both antigens and inflammatory cytokines to DCs for antigen cross-priming which activates cytotoxic CD8+ T lymphocytes [89–91]. Yatim et al. demonstrated that RIPK1 expression and NF-κB activation during programmed cell death are essential for initiating CD8+ T cell adaptive immunity and that CD8+ T cells activated by immune responses that necroptotic cells incurred released various effector cytokines, demonstrating in vivo cytolytic effects and defended mice against tumorigenesis [89]. Werthmöller et al. reported that the use of a combination of the pan-caspase inhibitor zVAD-fmk, which has been demonstrated to induce necroptosis, and other therapeutics, including radiotherapy, chemotherapy and hyperthermia, for the treatment of melanoma remarkably reduced tumor growth by reducing the tumor infiltration of regulatory T cells (Tregs) and increasing DC and CD8+ T-cell infiltration in the tumor microenvironment [24]. Schmidt et al. [92] showed that necroptotic cervical cancer cells induced by PolyI:C, which is a viral dsRNA analog that triggers necroptosis in cervical cancer cells, produced interleukin-1α (IL-1α), which is essential for the activation of DCs to release IL-12, which is a cytokine pivotal for antitumor effects, and that the expression level of RIPK3 in cervical carcinoma cells may predict the efficacy of PolyI:C-induced immunotherapy; therefore, immunotherapeutic treatment should be customized according to the RIPK3 level.

**Fig. 2:**
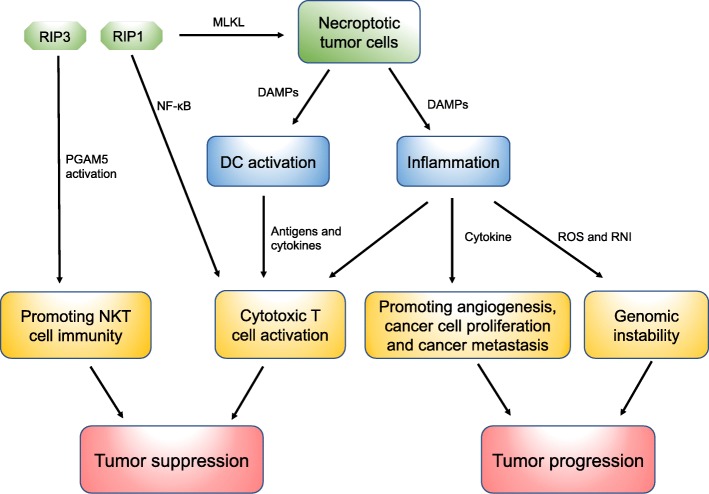
Role of necroptosis in cancer immunity

Despite the role of necroptosis in the induction and amplification of cancer immunity, multiple lines of evidence indicate that the immune inflammatory cells recruited by necrosis/necroptosis can promote tumor development by fostering angiogenesis, promoting cancer cell proliferation, and accelerating cancer metastasis [1, 93]. Additionally, necrotic/necroptotic cells can release regulatory cytokines, such as IL-1α, which can directly stimulate the proliferation of neighboring cells and potentially facilitate neoplastic progression [1, 93]. Activated inflammatory cells may also release reactive nitrogen intermediates (RNI) and ROS that can damage DNA and lead to genomic instability, thereby facilitating tumorigenesis [93] (Fig. 2).

Through the release of DAMPs into the tissue microenvironment, necroptotic tumor cells may provide both antigens and inflammatory cytokines to DCs for antigen cross-priming which activates cytotoxic ﻿CD8+ T lymphocytes, resulting in tumor cell elimination. However, DAMPs released by necroptotic cells may also recruit immune inflammatory cells and incur inflammation, which can promote tumor development by fostering angiogenesis, promoting cancer cell proliferation, and accelerating cancer metastasis. Activated inflammatory cells may also release reactive nitrogen intermediates (RNI) and ROS that can damage DNA and lead to genomic instability, thereby facilitating tumorigenesis. Moreover, RIPK1 expression and NF-κB activation during programmed cell death are essential for initiating CD8+ T cell adaptive immunity, and RIPK3 has been suggested to regulate NKT cell function and promote the NKT cell-mediated anti-tumor immune response by activating mitochondrial phosphatase phosphoglycerate mutase 5 (PGAM5).

Necroptosis has also been shown to generate an immunosuppressive tumor microenvironment in vivo in PDA and, thus, promote the oncogenesis of pancreatic cancer [64]. In RIPK3 knockout p48^Cre^;Kras^G12D^ pancreases, the percentages of B cells and T cells were elevated, the percentages of peritumoral myeloid-derived suppressor cells (MDSCs) and tumor-associated macrophages (TAMs), both of which can not only inhibit antitumor immune reactions but also stimulate tumor growth and metastasis [94, 95], were reduced and the expression of programmed death-ligand 1(PD-L1), a ligand which negatively regulates T cell antigen receptor signaling through interacting with its receptor PD-1 [96], in macrophages was decreased. Furthermore, the blockade of necroptosis may expand and activate T cells, which is a promising avenue for ameliorating the unsatisfactory efficacy of checkpoint-based immunotherapeutics in pancreatic cancer [64].

### Mechanisms of necroptosis promoting tumor progression and the role of necroptosis in Cancer metastasis

As mentioned above, necroptosis has been shown to perform antitumor functions in cancer; however, mounting evidence suggests that as a pathway triggering inflammatory responses, necroptosis may also play a tumor- promoting role, suggesting that the necroptosis pathway is a double-edged sword in cancer.

For instance, a study conducted by Liu et al. [17] demonstrated that in several breast cancer cell lines, the knockout of the RIPK1, RIPK3, or MLKL genes in cancer cells markedly reduced their tumorigenicity and appeared to sensitize breast cancer cells to radiotherapy. Moreover, in a xenograft model, the necroptosis inhibitor NSA (necrosulfonamide) greatly delayed tumor growth [17]. Additionally, the authors also reported that the higher phosphorylation levels of MLKL were correlated with a poorer prognosis and shorter survival in human patients with colon and esophageal cancer, indicating that necroptotic genes play a critical role in tumor promotion [17]. Furthermore, Seifert et al. [64] reported that the in vivo deletion of RIPK3 or RIPK1 attenuated tumor progression and immunosuppression in mice and explained these results by suggesting that necroptosis promoted pancreatic oncogenesis because CXCL1 (chemokine (C-X-C motif) ligand 1), which is a chemokine attractant, and Mincle signaling induced by necroptosis promotes the induction of adaptive immunosuppression by myeloid cells. Collectively, these studies indicate that the necroptosis pathway may increase the risk of tumor progression. The mechanisms underlying this seemingly paradoxical phenomenon may be related to the inflammatory response triggered by necroptosis, which may provide a tumor- promoting inflammatory microenvironment or elevated reactive oxygen species (ROS) production, which is correlated with genomic instability [97], ultimately accelerating malignant transformation and cancer progression [98, 99].

Metastasis is the primary cause of resultant mortality in cancer patients and involves the dissemination of cancer cells from the primary site to distant organs through the circulatory system.

The role of necroptosis in metastasis also exhibits duality. Fu et al. reported that in an in vivo osteosarcoma model, not only primary tumors but also lung metastases were markedly reduced by shikonin, which is a component used in Chinese herbal medicine, probably by inducing RIPK1- and RIPK3-dependent necroptosis [100]. One possible mechanism underlying the antimetastatic role of necroptosis may be its function in the regulation of ROS production, which involves necroptosis in extracellular matrix (ECM) detachment and metabolism and, ultimately, in cancer metastasis [101]. Indeed, RIPK3 has been demonstrated to regulate the production of downstream ROS [101, 102] and could activate multiple metabolic enzymes to modulate TNF-induced ROS production [51] during the process of necroptosis, which jointly induces a considerable production of ROS, thus enabling necroptosis to kill metastatic cancer cells by incurring ROS bursts. Accordingly, necroptosis may be a critical pathway inhibiting tumor metastasis.

However, contrasting evidence indicates that under certain circumstances, necroptosis may promote cancer cell metastasis. Extravasation, which is the process of tumor cell exit from the blood vessels and entry into a secondary site, is a crucial step in metastasis. Strilic et al. reported that tumor cells can induce necroptotic endothelial cell death to promote tumor cell extravasation and cancer metastasis via the activation of DR6 (death receptor 6) [63]. That study demonstrated that when cocultured with tumor cells, endothelial cells undergo necroptotic cell death. Similarly, after treatment with metastatic tumor cells, murine lung epithelial cells demonstrated necroptotic features. Moreover, the binding of DR6 to its ligand APP (amyloid precursor protein) promoted endothelial cell death and cancer cell extravasation. Strilic et al. explained that endothelial cells subjected to necroptotic death provide a tunnel through which tumor cells can pass and start to extravasate and/or the DAMP (damage-associated molecular pattern) molecules generated by necroptotic cells exert effects on tumor cells and adjacent endothelial cells, thus promoting the extravasation and metastasis of cancer cells. Thus, the authors suggested that therapies targeting DR6-mediated endothelial cell necroptosis may represent a new approach for preventing cancer metastasis.

In summary, the net effect of necroptosis on oncogenesis and cancer metastasis remains undefined because the specific role of necroptosis may not be universal and may vary according to the different biological traits or tumor microenvironments of each cancer type. Whether necroptosis facilitates or suppresses tumor growth and metastasis cannot be conclusively determined.

### Necroptosis and Cancer subtypes

Recently, Seehawer et al. coincidently found that of the two major subtypes of liver cancer, i.e., hepatocellular carcinoma (HCC) and intrahepatic cholangiocarcinoma (ICC), the type that animal models develop is determined by the gene-delivery technique, i.e., HCC via hydrodynamic tail-vein injection (HDTV) and ICC via in vivo electroporation, and that the cancer-promoting genes that they transferred were the same [6].

These confounding results were deciphered by the following findings: HDTV triggered apoptosis in the microenvironment, whereas electroporation triggered necroptosis, and the electroporated livers showed higher levels of ﻿phosphorylated MLKL and elevated mRNA expression of RIPK3 [6], which are biomarkers of necroptosis. The authors explained that necroptotic cells may release DAMPs that can shape the microenvironment via cytokines released by immune cells with pattern recognition receptors (PRRs) and that the necroptotic microenvironment may direct the lineage commitment of liver cancer, causing the switch from HCC to ICC development; this process is independent of the oncogenic drivers but may be involved in the epigenetic regulation of the genes Tbx3 and Prdm5 [6]. Additionally, the pharmacological or genetic inhibition of necroptosis reverts the necroptosis-dominated microenvironment and converts ICC to HCC [6], which further substantiates the role of necroptosis in determining liver cancer subtypes. The study provides a revolutionary insight into how tumor microenvironment may be shaped by a specific cell death modality and may eventually regulate ﻿lineage commitment in liver tumorigenesis and thus determine cancer subtypes, and further investigations are warranted to explore the role of necroptosis on other types of cancer and the fundamental mechanisms behind.

### Necroptosis in Cancer therapies

Inducing and/or manipulating necroptosis in anti-cancer therapies represent a promising therapeutic approach for bypassing acquired or intrinsic apoptosis-resistance, serving as an alternative way to eliminate apoptosis- resistant cancer cells. A growing arsenal of compounds and multiple chemotherapeutic agents have been reported to trigger necroptosis in cancer cells (Table 3).

**Table 3:** Compounds that induce necroptosis in cancer therapy

Compounds and Agents	Category	Mechanisms of Necroptosis Induction	Cancer Type	Reference
Shikonin	naphthoquinone	ROS production; RIPK1/RIPK3 necrosome formation	leukemia; osteosarcoma; pancreatic cancer; glioma	[100, 104, 103, 105]
Staurosporine	alkaloid	RIPK1/MLKL dependent	leukemia	[106, 107]
Neoalbaconol	albatrellus confluens extract	autocrine secretion of TNFα; remodeling cellular energy metabolism	nasopharyngeal carcinoma	[108, 109]
Resibufogenin	bufadienolide	upregulating RIPK3 and MLKL protein	colorectal cancer	[110]
Radiotherapy	radiation	inhibition of caspase-8 activation	thyroid cancer; adrenocortical cancer; colorectal cancer cells; glioblastoma	[111]
5-FU	chemotherapeutic agent	TNF-α production; RIPK1 activation	colorectal cancer	[114]
B12536	polo-like kinase inhibitor	leading to mitotic catastrophe	prostate cancer	[115]
Compound C	AMP-activated protein kinase inhibitor	Calpain/Cathepsin-mediated	glioma	[116]
Sorafenib	multikinase inhibitor	ROS production; RIPK1 activation	multiple myeloma; Hodgkin’s lymphoma	[117]
Aurora Kinase A Inhibitor	Aurora Kinase Inhibitor	facilitating necrosome activation	pancreatic cancer	[120]
TRAIL	death receptor ligand	TNFR1 signaling; RIPK1/RIPK3 dependent;ROS production	colon cancer; liver cancer; pancreatic cancer	[121, 122]
CD95L	death receptor ligand	CD95 signalling; regulation of cIAPs	pancreatic cancer	[123]
Oncolytic viruses	virus	exposure of immunogenic molecules	glioma; ovarian cancer	[124]
Hemagglutinating virus	virus	calcium-calmodulin kinase II dependent	neuroblastoma	[127]
Silver nanoparticles	metal nanoparticle	RIPK1/RIPK3/MLKL dependent	pancreatic cancer	[128]
Selenium nanoparticles	metal nanoparticle	RIPK1 dependent	prostate cancer	[131]
Smac mimetics	IAP antagonist	ROS production; cIAP inhibition; TNFα dependent	leukemia; pancreatic cancer	[132]
MG132 and bortezomib	proteasome inhibitors	RHIM-dependent	leukemia	[135]
Obatoclax	Bcl-2 inhibitor	Atg5-dependent necrosome assembly on autophagosomes	rhabdomyosarcoma; ALL	[136]
PolyI:C	viral dsRNA analog	RIPK3 dependent; TLR3/TLR4 activation	cervical cancer; colon cancer	[92]
ZZW-115	NUPR1 inhibitors	inducing mitochondrial metabolism rupture	pancreatic cancer	[137]

## Natural compounds

Shikonin, which is a naturally occurring naphthoquinone, was the first reported small molecule to induce necroptosis, and shikonin-induced necroptosis was found to bypass the resistance to cancer drugs mediated by drug transporters or antiapoptotic Bcl-2 proteins in human leukemia cell lines [138]. Subsequent studies have suggested that multiple shikonin analogs could also bypass drug resistance via the induction of necroptosis [41]. Fu et al. [100] reported that in osteosarcoma models, both the size of the primary tumor and metastasis to the lung were significantly reduced by shikonin and that the OS in the model with lung metastasis was increased. These findings highlight the profound antitumor role played by shikonin in both primary and metastatic sites in osteosarcoma, and this role is likely mediated by RIPK1- and RIPK3- dependent necroptosis. Recently, Chen et al. reported that in addition to inducing apoptosis, shikonin could induce necroptosis in pancreatic cancer by modulating RIPK1 and RIPK3 expression [103]. ﻿Shikonin also reportedly provoked mitochondrial ROS production of triple negative breast cancer cells, which disrupted breast cancer cells either by necroptosis or apoptosis [104]. Analogously, shikonin was demonstrated to cause cell death in glioma cells via the induction of necroptosis [105].

Staurosporine (STS), which is an alkaloid originally extracted from the bacterium Streptomyces staurosporeus [139, 140], has long been used in vitro to trigger apoptosis in many different cell types [106] and has been reported to induce RIPK1 and MLKL-dependent necroptotic cell death in leukemia cells when caspase activation is compromised [107]. The enzymatic role of poly(ADP-ribose)polymerase (PARP) was found to be dispensable for STS-induced necroptosis [107].

Neoalbaconol (NA), which is a constituent isolated from the fungus Albatrellus confluens, was reported to induces necroptosis by remodeling cellular energy metabolism in cancer cells [108]. NA has been shown to initiate necroptosis by promoting the autocrine secretion of TNFα via the regulation of the RIPK/NF-κB signaling pathway and RIPK3-dependent ROS production [109].

Resibufogenin, a member of bufadienolide family, is a bioactive compound extracted from toad venom [141]. It has been shown to exhibit anti-proliferative effects in multiple cancer cells [142–144]. Recently, Han et al. [110] have reported that resibufogenin was shown to suppress the growth and metastasis of colorectal cancer by inducing RIPK3 necroptosis both in vitro and in vivo ﻿through upregulating RIPK3 and MLKL protein at Ser358. The study also demonstrated that resibufogenin may activate ﻿three key metabolic enzymes, including glycogen phosphorylase (PYGL), glutamine synthetase (GLUL), and glutamate dehydrogenase (GLUDl) in a RIPK3 dependent manner, and resibufogenin was also found to suppress liver metastasis of colorectal cancer in mouse models [110].

## Radiotherapy and chemotherapeutic agents

In addition to natural compounds that induce necroptosis, radiation and chemotherapy can trigger necroptotic cell death [111–113].

In anaplastic thyroid cancer (ATC) and adrenocortical cancer (ACC) cell subjected to radiotherapy, in addition to apoptosis, necroptotic cell death was demonstrated to play a role in the induction of cell demise [111]. The RIPK1 inhibitor Nec-1 and caspase inhibitor zVAD synergistically increased cellular survival as the dosage of radiotherapy increased, highlighting the crucial role of apoptosis and necroptosis in the radiation-induced cell death of ATC and ACC [111]. This study indicated that pronecroptotic agents might enhance the antitumor effects of radiotherapy while reducing the dosage of radiation and attendant damage. In human colorectal cells, radiotherapy in combination with hyperthermia has been found to trigger necroptosis [113]. Moreover, in glioblastoma, high doses of radiation may inhibit the activation of caspase-8, leading to the necrosome formation, and thus, necroptosis is executed, but in response to low-dose radiation, active caspase-8 induces apoptosis [112].

Regarding chemotherapeutic agents, necroptotic cell death was identified as a vital mechanism of antitumor activity mediated by 5-FU. Pan-caspase inhibitors were found to facilitate TNF-α-dependent necroptosis induced by 5-FU. In an in vivo colorectal cancer xenograft model, a pan-caspase inhibitor was found to synergize with 5-FU to suppress tumor growth [114].

## Kinase inhibitors

Necroptosis has been found to be involved in the antitumor role of various kinase inhibitors.

Bl2536, which is a small molecule inhibitor of the mitotic kinase polo-like kinase 1 (Plk1), was demonstrated to inhibit Plkl during mitotic progression and lead to mitotic catastrophe, resulting in necroptotic cell death in androgen-insensitive prostate cancer cells [115].

Compound C, which is also named dorsomorphin, is a small molecule widely used as a selective AMP-activated protein kinase inhibitor and has been reported to kill glioma cells by multiple mechanisms, including autophagy and necroptosis [116].

Under conditions of inefficient caspase activation, sorafenib, which is a multikinase inhibitor, induces cell death by necroptosis in multiple myeloma cells [117]. Furthermore, the combinatorial treatment of sorafenib and the histone deacetylase inhibitor Givinostat was shown to synergistically induce ROS-dependent necroptosis, which mediated the anti-tumor effects, in relapsed/refractory Hodgkin’s lymphoma cell line xenografts [118]. In addition, sorafenib has been suggested to kill cancer cells by necroptosis during defective or inefficient autophagy, which may cause the accumulation of p62 protein levels, which may serve as a signaling platform for the initiation of necroptosis when undergoing cellular damage or stress. In contrast, sorafenib was demonstrated to protect acute lymphoblastic leukemia cells from necroptosis induced by multiple necroptosis- inducing drugs [119].

Aurora kinase A inhibitor, whose antitumor activity has been wide reported [145], was shown to markedly inhibit PDA growth both in vitro and in vivo via the induction of necroptosis by inhibiting Aurora kinase A (AURKA), which was found to inhibit the activation of necrosome [120].

## Death receptor ligand

TRAIL (tumor necrosis factor (TNF)-related apoptosis inducing ligand) is a death receptor ligand that reportedly induces necroptosis instead of apoptosis in colon and liver cancer cells under conditions of acidic extracellular pH, and PARP-1 is an active effector downstream of RIPK1/RIPK3 initiators [121]. TRAIL was also found to induce necroptosis in human pancreatic cancer cells, which is regulated by ROS and caspase-9/− 2 [122].

CD95 Ligand (CD95L), which is also known as Fas ligand, is a death receptor ligand known to induce apoptosis upon binding its receptor, i.e., CD95. CD95L has been reported to induce necroptosis upon the downregulation of cIAPs [123], which polyubiquitinate RIPK1 to steer the pathway towards NF-κB signaling [14]. A study conducted by Pietkiewicz et al. demonstrated that the combined treatment of CD95L and gemcitabine simultaneously induced apoptosis and necroptosis in pancreatic carcinoma cells and that gemcitabine significantly switched CD95- induced cell death into necroptosis when combined with CD95L in pancreatic carcinoma cells [146].

### Viruses

Oncolytic viruses (OVs) are novel anticancer agents whose antitumor activities are attributed to oncolysis and induced antitumor immunity [147]. OVs induce mostly immunogenic cancer cell death (ICD), which includes necroptosis, through exposure to calreticulin and the release of ATP, high-mobility group box 1 (HMGB1), DAMP, and PAMP, which may activate dendritic cells and incur adaptive antitumor immunity [124, 147]. These viruses also encode certain genes to regulate ICDs, including necroptosis, which according to the authors, can be genetically modified to elicit a certain desired modality of ICD in the cancer cells infected by the viruses [124]. For instance, in ovarian cancer cells, vaccinia virus can cause necroptosis [125]. In glioma, ICD induced by newcastle disease virus occurred in a caspase-independent fashion and was blocked by nec-1, implicating the contribution of necroptosis [126].

Hemagglutinating virus of Japan-envelope (HVJ-E) also triggers necroptosis in xenograft model of human neuroblastoma cells, which is triggered by elevated level of cytoplasmic calcium activating calcium-calmodulin kinase II (CaMK II) [127].

## Metal nanoparticles

Silver nanoparticles (AgNPs) are metal nanoparticles known to induce apoptosis in cancer cells [128] that have gained increasing attention as promising anticancer agents because of their unique abilities to penetrate cell membranes and easily cross biological barriers [129, 130]. According to a study conducted by Zielinska et al., in PANC-1 cells, AgNPs could induce both necroptosis and apoptosis and substantially increase the levels of both the tumor suppressor p53 protein and proteins related to necroptosis and autophagy, including RIP-1, RIP-3, MLKL and LC3-II. AgNPs were found to inhibit the proliferation and decrease the viability of PANC-1 cells; moreover, pancreatic cancer cells were markedly more sensitive than nontumor pancreatic cells to AgNP-induced cytotoxicity [129].

Selenium nanoparticles (SeNPs) were found to induce ROS-mediated necroptosis in a prostate adenocarcinoma cell line, which was observed to be dependent on RIP1 but was found not to require RIP3 and MLKL activation and, thus, independent of necrosome formation [131].

Other metal nanoparticles, such as ZnO, have also been demonstrated to induce necroptosis in cancer cells, mostly leading to increased levels of ROS [148, 149].

## Other necroptosis inducers

### Smac mimetics

Smac mimetics are small molecule mimics of second mitochondrial activator of caspases (Smac), which is an endogenous protein that promotes apoptosis by inhibiting cIAPs [19]. Smac mimetics have been recognized as emerging anticancer agents [150]. Similar to shikonin, a Smac mimetic was reported to permit cells to bypass apoptosis resistance via the necroptotic pathway [132]. Laukens et al. demonstrated that in leukemia cells, a Smac mimetic augmented TNFα-induced cell death via necroptosis in the absence of FADD or caspase-8 in apoptosis-resistant cells or via apoptosis in apoptosis-proficient cells, suggesting that Smac mimetics may be developed as novel chemotherapeutics to promote necroptosis as an alternative programmed cell death process to overcome apoptosis resistance [132]. Furthermore, ROS were found to be required for the regulation of Smac mimetic/TNFα-induced necroptotic signaling, specifically in the enhancement of RIPK1/RIPK3 necrosome stabilization when stimulated by a Smac mimetic/TNFα [133]. Recently, Hannes et al. [134] reported that the Smac mimetic BV6 alone or combined with TNFα could induce necroptosis in pancreatic cancer cells in which apoptosis was blocked by inducing the formation of the RIPK1/RIPK3 necrosome.

### Proteasome inhibitors

The ubiquitin-proteasome system is the major mechanism by which cells selectively degrade unneeded or damaged cellular proteins by proteolysis and thus, has an essential role in many cellular processes including cell cycle and cell demise [151]. Proteasome inhibitors have therefore been deemed as a promising anti-cancer agent clinically. For instance, a classic proteasome inhibitor, bortezomib, has been a success in treating multiple myeloma [152]. Recently, Moriwaki et al. [135] reported that ﻿proteasome inhibitors MG132 and bortezomib can trigger the RIPK3/MLKL dependent necroptosis in both fibroblasts in mouse models and human leukemia cells. Notably, proteasome- inhibitor- induced necroptotic pathway is independent of caspase inhibition, yet still requires intact RIP homotypic interaction motif (RHIM) [135]. The study has indicated that the ubiquitin-proteasome system may be a potential regulatory pathway for RIPK3-dependent necroptosis and that proteasome inhibitors can be developed as an anti-cancer agent targeting necroptotic pathway.

### Obatoclax

Obatoclax (GX15–070), which is a small-molecule Bcl-2 inhibitor of antiapoptotic Bcl-2 proteins, was recently reported to trigger necroptosis by assembling the necrosome onto autophagosomes; this process links the induction of autophagy to cell death via necroptosis [136].

### PolyI:C

Polyinosinic:polycytidylic acid (PolyI:C), which is a viral dsRNA analog, was reported to trigger necroptosis in cervical cancer, which strictly depended on the expression of RIPK3. Necroptotic cervical cancer cells produce IL-1α, which was essential for activating DCs to release IL-12, which is a cytokine crucial for antitumor activities [92]. In colon carcinoma cell lines, in addition to inducing immune or macrophage activation, PolyI:C can trigger necroptosis alone or combined with the pan-caspase inhibitor zVAD, supporting tumor retardation mediated by immune effectors in vivo [57].

### NUPR1 inhibitors

NUPRI1 is a member of intrinsically disordered proteins (IDPs), which engages in various cancer-related processes including cell-cycle regulation, apoptosis [153], cancer metastasis [154], DNA repair response [155] and etc. It has recently drawn remarkable attention because it has been shown to promote progression and development of pancreatic cancer [137], making it a promising anti-cancer therapeutic target. A newly synthesized NUPR1 inhibitor, named ZZW-115 was reported to inhibit the growth pancreatic xenografted tumors in vivo dose-dependently via the induction of necroptosis by inducing mitochondrial metabolism rupture, and there was no evidence of off-target effects [156]. The study suggested that ﻿ZZW-115 is a promising therapeutic agent for treating various cancers due to its effectiveness in targeting NUPR1.

### Selectivity of Pronecroptotic therapy

Since necroptosis may also occur under various physiological conditions, one major concern about pronecroptotic therapy is whether necroptosis can be selectively induced in cancer cells yet cause no harm to normal cells. In fact, multiple agents and chemotherapeutic drugs that have been granted for marketing or in clinical trials have been recognized as selective necroptosis inducer in cancer cells in specific cancer types, including shikonin and its analogs [41, 157], TRAIL [16], obatoclax [158], metal nanoparticles [148] et cetera. The safety of those compounds and drugs in vivo have been verified, suggesting triggering necroptosis in cancer cells is not necessarily injurious to normal cells [159]. However, to further improve the selectivity of the agents and drugs targeting necroptosis, future therapeutics can combine necroptosis inducers to tumor-guiding agents or tumor-targeting antibodies.

## Conclusion and perspectives

Necroptosis is a necrotic programmed cell death with potent immunogenicity that engages in complex interplay with autophagy and apoptosis. Accumulating evidence suggests that necroptosis plays a vital role in the prognosis of cancer patients, cancer progression and metastasis, cancer immunosurveillance and cancer subtypes. Targeting necroptosis via various drugs, compounds and agents inducing or manipulating the necroptotic pathway has also emerged as a novel approach for bypassing apoptosis- resistance and supporting antitumor immunity in cancer therapy.

The general downregulation of the expression of key proteins in the necroptotic pathway suggested that cancer cells may also evade necroptosis to survive; however, in some types of cancer, such as PDA, the expression level of key mediators has been shown to be elevated. Necroptosis can induce robust immune responses through the release of DAMPs and various immunoregulatory cytokines that activate DCs and enhance antitumor immunity; however, the recruited inflammatory cells may foster angiogenesis and cancer invasiveness and generate an immunosuppressive tumor microenvironment. Necroptosis also reportedly promotes oncogenesis and cancer metastasis despite evidence of its antimetastatic role in cancer. In addition, necroptosis can direct lineage commitment to determine the cancer subtypes in certain cancers. A plethora of compounds and agents have been found to trigger or manipulate necroptosis and exhibit promising antitumor efficacy. ﻿However, most studies investigating the therapeutics targeting necroptosis are based on in vitro experiments and/or animal models, thus the feasibility of the clinical use of these compounds and anticancer agents still needs to be assessed in vivo and clinical trials. Further, the off-target effects of the necroptosis-targeting therapeutics should be scrutinized, and novel approaches that conjugate necroptosis inducers and tumor-guiding agents should be developed to enhance safety and selectivity.

In conclusion, the exact role of necroptosis in cancer remains to be fully elucidated. Although various reports support the antitumor functions of necroptosis, mounting evidence indicates that necroptosis also promotes tumor progression and metastasis, suggesting that the specific role of the necroptosis pathway in cancer should be contextualized in different types of cancers. The current knowledge and evidence are inadequate to determine whether necroptosis generally promotes or suppresses tumor cell growth and/or cancer metastasis. The diametrical conclusions drawn from different studies investigating the relevance of necroptosis in cancer may be attributed to the lack of specific markers of necroptosis, the pleiotropic role of necroptotic mediators, and the distinct tumor microenvironment of each type of cancer. Consequently, the discovery of a specific necroptosis marker for the identification of necroptosis, a thorough investigation of the molecular mechanism and physiological and pathological roles of necroptosis and a clarification of its crosstalk with other cell death machineries and its interaction with the immune system are urgently needed to decipher the mystery of the relevance of necroptosis in cancer and further develop antitumor therapeutics targeting.

## Abbreviations

ACC: Adrenocortical cancer
AgNPs: Silver nanoparticles
AML: Acute myeloid leukemia
ANT: Adenine-nucleotide translocators
APP: Amyloid precursor protein
ATC: Anaplastic thyroid cancer
ATP: Adenosine triphosphate
AURKA: Aurora kinase A
CaMK II: Calcium-calmodulin kinase II
cIAP: Cellular inhibitor of apoptosis protein 1
CLL: Chronic lymphocytic leukemia
CXCL1: Chemokine: C-X-C motif ligand 1
CYLD: The deubiquitinase cylindromatosis
DAMP: Damage-associated molecular pattern
DC: Dendritic cell
DR6: Death receptor 6
ECM: Extracellular matrix
FADD: FAS-associated death domain protein
GLUD1: Glutamate dehydrogenase 1
GLUL: Glutamate-ammonia ligase
HCC: Hepatocellular carcinoma
HDTV: Hydrodynamic tail-vein injection
HMGB1: High-mobility group box 1
HR: Hazard ratio
ICC: Intrahepatic cholangiocarcinoma
ICD: Immunogenic cell death
IDPs: Intrinsically disordered proteins
IL-1α: Interleukin-1α
MDSC: Myeloid-derived suppressor cells
MLKL: Mixed lineage kinase domain-like pseudokinase
NA: Neoalbaconol
nec-1: Necrostatin-1
NF-κB: Nuclear factor kappa B
NK: Natural killer cell
NKT: Natural killer T cell
NSA: Necrosulfonamide
OS: Overall survival
OV: Oncolytic viruse
PARP: Poly(ADP-ribose)polymerase
PCD: Programmed cell death
PDA: Pancreatic ductal adenocarcinoma
PDH: Pyruvate dehydrogenase
PD-L1: Programmed death-ligand 1
PGAM5: Phosphoglycerate mutase 5
Plk1: Polo-like kinase 1
PolyI:C: Polyinosinic:polycytidylic acid
PRRs: Pattern recognition receptors
PYGL: Glycogen phosphorylase
RFS: Recurrence-free survival
RHIM: RIP homotypic interaction motif
RIPK: Receptor-interacting protein [RIP] kinase
RNI: Reactive nitrogen intermediates
ROS: Reactive oxygen species
S161: Serine residue 161
SeNPs: Selenium nanoparticles
Smac: Small molecule mimics of second mitochondrial activator of caspases
STS: Staurosporine
TAA: Tumor-associated antigen
TAMs: Tumor-associated macrophages
TCRs: T cell receptors
TEM: Transmission electron microscopy
TLR: Toll like receptor
TNFR: Tumor necrosis factor receptor
TRADD: TNFR-associated death domain
TRAF2: TNFR-associated factor 2
Treg: Regulatory T cell
TSA: Tumor-specific antigen-or
WB: Western blot
